# Transition to parenthood in the neonatal care unit: a qualitative study and conceptual model designed to illuminate parent and professional views of the impact of webcam technology

**DOI:** 10.1186/s12887-017-0917-6

**Published:** 2017-07-11

**Authors:** Susan Kerr, Caroline King, Rhona Hogg, Kerri McPherson, Janet Hanley, Maggie Brierton, Sean Ainsworth

**Affiliations:** 1 School of Health & Life Sciences, Glasgow Caledonian University, Cowcaddens Road, Glasgow, G4 OBA Scotland; 2 National Health Service (NHS) Greater Glasgow & Clyde, West House, Gartnavel Royal Hospital, 1055 Greater Western Road, Glasgow, G12 0YN Scotland; 3 School of Health and Social Care, Edinburgh Napier University, Sighthill Campus, Sighthill Court, Edinburgh, EH11 4BN Scotland; 4 Victoria Hospital, NHS Fife, Hayfield Road, Kirkcaldy, KY2 5AH Scotland

**Keywords:** Neonatal unit, Technology, Perceptions, Parents, Professionals, Qualitative

## Abstract

**Background:**

Complications during pregnancy, childbirth and/or the postnatal period may result in the admission of a baby to a neonatal unit (NNU). While the survival and long-term prospects of high-risk infants are enhanced by admission, the enforced separation of the parent and child may have psychological consequences for both. There is a need to develop and evaluate interventions to help parents ‘feel closer’ to their infants in circumstances where they are physically separated from them. In this paper we present findings from an in-depth, theoretically-driven, evaluation of a technological innovation designed to address this need. The study sought to explore parent and professional views of the impact of the technology, which transmits real-time images of the baby via a webcam from the NNU to the mother’s bedside in the post-natal care environment.

**Methods:**

A qualitative approach was adopted, guided by a critical realist perspective. Participants were recruited purposively from a NNU located in East-central Scotland. Thirty-three parents and 18 professionals were recruited. Data were collected during individual, paired and small group interviews and were analysed thematically. Following the initial analysis process, abductive inference was used to consider contextual factors and mechanisms of action appearing to account for reported outcomes.

**Results:**

Views on the technology were overwhelmingly positive. It was perceived as a much needed and important advancement in care delivery. Benefits centred on: enhanced feelings of closeness and responsiveness; emotional wellbeing; physical recovery; and the involvement of family/friends. These benefits appeared to function as important mechanisms in supporting the early bonding process and wider transition to parenthood. However, for a small number of the parents, use of the technology had not enhanced their experience and it is important, as with any intervention, that professionals monitor the parents’ response and act accordingly.

**Conclusions:**

With a current global increase in premature births, the technology appears to offer an important solution to periods of enforced parent-infant separation in the early post-natal period. The current study is one of a few world-wide to have sought to evaluate this form of technology in the neonatal care environment.

## Background

Complications during pregnancy, childbirth and/or in the postnatal period may result in the admission of a baby to a neonatal care unit (NNU). Recent estimates suggest that 8–12% of babies in the developed world receive some form of care in a NNU, with the most common reason for admission being premature birth [1, 2]. While the survival and long-term prospects of high-risk infants are enhanced by admission to the NNU, the separation of the mother and child, enforced by different care environments, can have psychological consequences for both [3].

“Transition to motherhood” has been described as a process of change that occurs as a woman begins to care for her child, to problem solve and to appraise herself as a mother [4, 5]. Mothers with infants admitted to a NNU are known to experience greater difficulties in transitioning to the maternal role than mothers with healthy babies [6, 7]. Reasons include periods of separation and reduced opportunities for early bonding that may lead to decreased levels of maternal responsiveness and sensitivity [8, 9]. Also, concern for the immediate and long-term health and development of their baby has been shown to result in higher levels of stress, anxiety and depression in mothers, which may persist after the baby has been discharged [9, 10]. Importantly, the psychological well-being of mothers is known to influence early parent-child interactions and can impact the social, emotional, behavioural and cognitive development of children in the short and longer term [9].

In Western societies, there have been significant changes in family structures and compositions in the past 30–40 years and this includes shifting expectations of the roles of fathers in the post-natal period [11]. As a consequence, the “transition” of fathers, as they seek to establish a relationship with the new baby and support their partner, has increasingly been explored [11]. While research on the psychological adjustment of fathers of children admitted to NNU is limited, elevated rates of depression and anxiety have been identified [12]. Also, similar to their partner, periods of enforced separation from their new-born baby can function as a barrier to the establishment of the early parent-child relationship [8].

A range of interventions designed to promote physical and emotional closeness between parents and their babies has been developed for use in NNUs. These interventions include: kangaroo care, which facilitates skin to skin contact; infant massage, designed to promote parent-child interaction; and, diaries written by staff on behalf of babies, which parents can access online [13–15]. While these interventions have been shown to be useful, further research is required to develop and evaluate interventions to help parents ‘feel closer’ to their infants when they are physically separated from them [9]. In recent years, interest has grown in the use of webcam technology to address this need, with this form of technology being used in the United States of America (USA), Australia, Singapore, the Netherlands [16–18] and most recently in Ireland [19] and the United Kingdom (UK) [20].

In this paper findings are presented from an in-depth evaluation of the impact of webcam technology used in a UK setting. The intervention, named *mylittleone*, involves an Internet Protocol (IP) camera being placed over a cot/incubator in the NNU, which transmits real-time images of the baby, wirelessly and securely, to a dedicated network hub, coupled to a tablet device kept by the mother in the post-natal care environment. To ensure confidentiality, the camera is fixed so that the images transmitted are of the individual cot/incubator and no sound is transmitted. The camera is switched off when nursing and/or medical procedures are undertaken, otherwise it is in constant operation and therefore allows a mother to view her baby whenever she wishes. The *mylittleone* technology was developed with the intention of promoting increased feelings of closeness, and, in turn, facilitating the parent-infant bonding process.

While technological innovations in the health field are generally introduced with laudable intentions, a critique of the literature suggests that the expected advantages are not a given and indeed there may be uncertainties or anxieties associated with their use [21]. Reactions appear to be influenced by a complex and inter-related array of social, psychological and technical factors, situated within the healthcare environment into which the new technology has been introduced [22]. For example, the introduction of new technologies may influence care expectations, re-define inter-personal relationships and/or impact feelings of agency [23]. It is therefore important that new technologies are evaluated to determine whether their anticipated benefits are realised and to identify any unanticipated consequences of their use. The views of those classified as ‘end users’ of the new product are particularly relevant in this regard. When considering the use of webcams in NNUs, while some important evaluative work exists, this has focused on implementation issues, including parental ‘satisfaction’ with the technology [16, 18], the impact on the workload of nurses [14, 15], and views of parents, nurses and doctors prior to the introduction of the technology [19]. To the best of our knowledge, there has, to date, been no theoretically driven, in-depth exploration of the impact of this form of technology on the parental role in the early postpartum period.

In light of the above, the aim of the current study was to explore parent and professional views of the impact of the *mylittleone* technology on the transition to parenthood and to uncover likely mechanisms of action. The study also sought to identify contextual factors that appeared to have influenced views of the technology.

## Methods

### Design

A qualitative approach, informed by a critical realist ontological perspective, was adopted [24]. In a critical realist study reality is considered to be largely, but not wholly, social constructed with the conditions and social relations involved in the production of knowledge (e.g. during a research interview) acknowledged as influencing its content [25, 26].

### Sample, setting and recruitment

Participants were recruited from a NNU located in east-central Scotland. At the time of recruitment this was the only NNU in the UK using the *mylittleone* technology.

Parents were recruited purposively [27] based on age, family size, socio-economic status, the medical condition of the baby and the length of stay/anticipated length of stay in the neonatal unit. Purposive sampling was used to ensure the views expressed were from a heterogeneous group of parents and thereby to enhance the potential transferability of the findings [27]. The only inclusion criterion was experience of using the *mylittleone* technology; there were no exclusion criteria. Staff working in the NNU distributed Study Information Sheets to mothers of the babies they were caring for; this included mothers who had been discharged (prior to their baby) and those who had not yet been discharged. Mothers who were interested in taking part, or who wished to receive further information before making up their minds, were asked to complete a form that gave permission for their contact details to be passed to the study researcher (CK). Participation was on a voluntary basis; parents were informed that if they did not wish to take part it would not affect their own or their baby’s care in any way. Those who agreed to participate were asked to invite their partner to take part in the study, if they wished. The aim was to recruit 30 parents, including fathers. All participants provided written consent.

Purposive sampling was also used to recruit members of the multi-disciplinary team of professionals caring for babies in the neonatal unit and parents in the postnatal care environment (e.g. neonatal nurses, midwives, doctors). The professionals were provided with an Information Sheet (distributed in the NNU and post-natal care environment by the study researcher) and were informed that participation was on a voluntary basis. The aim was to recruit c.20 professionals. Again, all participants provided written consent.

### Data collection

Data were collected from the parents during individual or paired semi-structured, face-to-face interviews (*n* = 25). Paired interviews were undertaken when both the mother and father had been recruited. The majority of the interviews (*n* = 17) were conducted in a private setting in the NNU, with eight conducted in the family home, as this was the parents’ preference. The professionals participated in individual, paired or small group face-to-face, semi-structured face-to-face interviews (*n* = 8), depending on their availability and/or preference. The decision to interview the professionals in this manner was largely pragmatic. The interviews with the professionals were conducted in a private location in the neonatal/postnatal care environment. Interview guides were used to facilitate the interview process and to ensure similar issues were addressed across the participant groups (see Table 1). The data were collected by the project researcher CK, between January and July 2015; the technology had been fully functional in the neonatal unit from November 2014. The researcher was unknown to the participants prior to the study commencing.

**Table 1:** Interview Guides (outline of content)

Parents	Professionals
Health during pregnancy	Own views and experience of *mylittleone* - expectations before introduced - reality of its use
Admission to NNU (anticipated/unanticipated)	Observations of parents’ experiences - general views/experience - any aspects of care made easier - any aspects of care made more difficult - partners/extended family
Experience of baby being cared for in NNU	- Impact on own caring role
Views and experience of *mylittleone* - general views/experience - any aspects of care made easier - any aspects of care made more difficult - views/experience of extended family	Other issues professionals wished to discuss
Other issues parents wished to discuss	

### Data analysis

The audio-recorded interviews were transcribed verbatim and checked for accuracy. Names and any identifying information were removed prior to the analysis. The data were analysed thematically, in NVivo version 10, using the process described by Braun & Clarke [28]. Familiarisation with the data was followed by a coding process that drew on a priori reasoning and was linked, deductively, to questions in the interview guide. The data were then indexed thematically (and inductively) based on what was discussed by the study participants. In the final stage, and in line with the critical realist approach underpinning the study [24], ‘abductive’ inference was used to consider the contextual factors and mechanisms of action that appeared to account for the reported outcomes [29]. This final stage in the analysis process was viewed through a theoretical lens informed by literature on the transition to parenthood [6, 7], parent-child bonding [30–32] and the experience of ‘new technologies’ in the health field [22, 33], with a conceptual model developed to explain the relationships.

The analysis was undertaken by the project researcher (CK). Other members of the research team (SK, RH, KMcP) provided peer-review and assisted with the interpretation of the data, to ensure rigour in the process.

### Ethical approval

The study was considered to be a service evaluation by the East of Scotland Ethics Committee (Ref: CYA/AG/13/GA/127) and so did not require NHS ethical approval. Ethical approval was therefore sought and granted from the School of Health & Life Sciences Ethics Committee at Glasgow Caledonian University.

## Results

### Participant characteristics

Thirty-three parents were recruited (25 mothers and 8 fathers). A profile of the participants is presented in Table 2. As noted, the age of the parents ranged from 19 to 44 years, with the majority being married or living with a partner. Eighteen of the babies (including one set of twins) were born before 37 weeks’ gestation. Those born after 37 weeks were most commonly admitted to the neonatal unit because they were jaundiced, had a suspected infection, breathing difficulties, low blood sugar, and/or because they were of low birth weight.

**Table 2:** Parent participants and their babies (*n* = 33)

Mother’s age (*n* = 25)	Frequency
19 years and younger	1
20–29 years	13
30–39 years	10
40 years and older	1
Range 18–44 years	
Father’s age (*n* = 8)	
19 years and younger	1
20–29 years	5
30–39 years	2
Range 19–39 years	
Marital Status	
Married/living with partner	22
Single	3
Baby’s gestation at birth in completed weeks	
< 28 weeks	2
28 < 32 weeks	6
32 < 37 weeks	9
37 + weeks	8
Range 26 weeks–41 weeks	
Baby’s sex	
Female	16
Male (includes 1 set of twins)	10
Other children	
Yes	14
No	11
Scottish Index of Multiple Deprivation (SIMD)^a^	
1–2	10
3	9
4–5	6

^a^[49] Participants’ postcodes were used to calculate scores. Areas scoring 1 are the most deprived; areas scoring 5 are the least deprived

Eighteen professionals were recruited. A profile of the professional participants can be found in Table 3. The professionals were neonatal nurses, midwives, nursery nurses and doctors.

**Table 3:** Professional participants (*n* = 18)

Place of work	Frequency
Neonatal Unit	10
Postnatal Ward	8
Professional Background	
Neonatal Nursing	8
Midwifery	3
Nursery Nursing^a^	5
Medicine	2
Gender	
Female	17
Male	1

^a^In the UK, neonatal nursery nurses (who have undertaken certified training) are responsible for the care and daily living needs of babies from admission to discharge, under the guidance of the nurse manager. Tasks include bathing, nappy changing, observations and providing parents with advice and support

The themes that emerged from the analysis of the data are presented under three broad categories: Benefits of *mylittleone*; Potential disadvantages of *mylittleone*; and, Extending *mylittleone’s* reach to the home environment. When presenting the findings, the accounts from the parents and professionals have been integrated, where appropriate, to allow similarities and differences in the views and experiences of the participant groups to be highlighted. The conceptual model is presented in the Discussion, with the findings considered, in light of extant empirical and theoretical literature.

### Benefits of *mylittleone*

The majority of the participants believed the development and deployment of *mylittleone* was an important advancement in the provision of neonatal care. The manner in which the technology had enhanced the parents’ experience was central in the accounts of both the parents and professionals. Themes that emerged from the data included: Being present when you’re not; Keeping mums (and dads) on an even keel; Helping mums to take care of themselves; and, “Showing off” the new baby.

#### Being present when you’re not

Mothers in the post-natal care environment most commonly have their babies with them immediately following the birth and throughout their hospital stay. However, when a baby is admitted to a neonatal care unit, periods of separation result and, depending on the mother’s physical health, these periods can be lengthy. The impact of separation from their child was discussed at length in the interviews, by both the mothers and fathers and, as exemplified in the following quotes, *mylittleone* was reported as helping them ‘feel closer’ to their babies when they could not be with them:

*[Mother 1; baby 6 weeks premature] Oh, it was brilliant, absolutely brilliant … I was so tired, that I couldn’t sit for longer than half an hour [in neonatal care] … I didn’t feel well, I was sick and I was dizzy, so when I got up the stairs [to post-natal ward], I put [mylittleone] right next to the bed, and even though I fell asleep pretty quick, it was like … it was like she was near me, because I could see her. Instead of being completely cut off from her, she was still there.*

[*Mother 12; baby 4 weeks premature] [It’s hard] when you’ve not got your baby with you, when everybody else does.*

*[Father 12] [But] you actually feel like you’re with them basically because you’re getting a live feed.*

*[Mother 12] Yes, so you’re present even when…*

*[Father 12] Even when you’re not.*

In addition to the increased feeling of proximity, there was a belief that *mylittleone* helped the mothers, in particular, to be more responsive to their baby’s needs and this included responding physically through the production of breast milk. As indicated below, mothers who had had previous experience of the neonatal environment were able to make useful comparisons.

*[Mother 2; baby 8+ weeks premature] It’s so different this time. With my little boy [also admitted to a neonatal unit] I couldn’t see him straightaway … whereas this time having the mylittleone camera … I can see her constantly, she’s right beside my bed, really. [Also], I’ve found with things like expressing milk, I’ve found that a lot easier.*

*[Professional interview 5, post-natal ward] I think it’s excellent … it’s like [the mothers] are really close to their babies … A lot of the mums…when the baby’s upset will go down the nursery. You know, they’ll say, the baby’s really upset I’m away down to see if it’s needing fed or what’s wrong with it. So, they’ll toddle away down and see.*

The feelings of ‘closeness’ and ‘responsiveness’ that *mylittleone* engendered appeared to be important in facilitating the process of ‘transitioning to parenthood’, and relatedly, to encourage the early bonding process between the parents (both mothers and fathers), and their babies, which, from what was described, would have been more challenging had the physical separation not been bridged by the technology.

#### Keeping mums (and dads) on an even keel

There was much discussion of the positive emotions associated with parents seeing their baby for the first time using the *mylittleone* technology.

*[Mother 24; baby 15+ weeks premature] You couldn’t wipe the smile off [husband’s] face when the doctor brought the tablet round.*

*[Father 24] It was like an overwhelming thing. I was just so happy and proud that I could see her, if that makes sense.*

*[Mother 2; baby 8+ weeks premature] As soon as the doctor [gave me the tablet] … I couldn’t speak, I just kept crying … it was amazing just to see this tiny little baby that you knew was yours, but there she was.*

The parents also discussed how the technology had enabled them to keep more stable emotionally in the days following the delivery. A key benefit appeared to be the reassurance connected with being able to see their baby was ‘okay’, that is, there had been no worsening of their condition. The comfort associated with this is clear in the narratives below.

*[Mother 1; baby 6 weeks premature] I’m not just saying this, but if I didn’t have the camera … I think I would have cracked up, because it’s been…I’ve been waking up during the night and kind of looking and being able to see that she was there and that she was sleeping, and I would be able to fall back asleep again.*

*[Father 19; baby full-term] It provides reassurance. You can see her snoozing and, as long as you can see the quilt going up and down, you know she’s breathing okay. We don’t fret when we are away from her because we can see her.*

Discussions of emotions extended to the low emotional state, commonly referred to as the ‘baby blues’, that mothers may experience a few days after the birth of their baby.

*[Mother 16, baby 5 weeks premature, also low birth weight] Everyone was kind of saying I would have a ‘baby blue day’ and I think I didn’t get that because I knew she was okay and I was able to see her … So I was waiting on that and I think that having [mylittleone] and watching her probably helped quite a bit, I didn’t ever have down days … I don’t quite know what I would’ve done without it.*

Also, seeing their baby on the tablet device allowed parents to prepare themselves emotionally for visiting the neonatal unit for the first time.

*[Mother 23; baby 9+ weeks premature] He was born at 11 min past four and my boyfriend went to see him about five and then brought [mylittleone] straight down, and I got to come up at about 11 to see him, so it was really good because I got to prepare myself by looking on the screen of what I was coming up to see, like the tubes and stuff, so it was quite nice.*

The continuous ability to monitor their baby’s progress was also reported by some as giving them ‘hope’ in terms of the immediate and longer-term health and development of their child. The baby referred to in the quote below had had a serious respiratory problem at birth and this had required intensive and prolonged medical interventions.

*[Father 21; baby full-term] Just little things isn’t it [to mother]? Because where we are it’s little steps, really little steps at a time, just like her hand moving and she’s trying to grab things and you can see.*

*[Mother 21] Or she’s trying to touch her face or things like that … It’s just these things that’s giving us that wee bit of hope.*

Finally, staff in the neonatal care unit stressed the almost ‘gift like’ quality of *mylittleone*, something which again appeared to enhance the emotional well-being of the parents.

*[Professional interview 8; neonatal unit] You get such a great reaction the minute the baby’s stable ... if I’ve had to take the [tablet] round, the mum’s just so happy, you know, she knows she wasn’t going to see her baby for [maybe] another 12 h and you have appeared and said, here she is here and you can watch her*.

From what was described, the positive impact on the parents’ emotional well-being appeared to be an important outcome associated with their use of *mylittleone*. Use of the technology allowed them to feel more connected to their baby, monitoring their welfare and progress, and thus to function in a parenting role, despite periods of separation imposed by the location of the baby in the NNU. Also, the ability to view their baby on the tablet device, in what they commonly perceived as the ‘alien’ environment of the neonatal unit, was considered important as it allowed parents to prepare themselves for an unanticipated transition, that of being the parent of a sick and/or premature baby.

#### Helping mums to take care of themselves

Another benefit related to the scope *mylittleone* provided in assisting the physical recovery of the mothers following the birth of their baby. The main issues discussed were sleep, rest, nourishment and the reduction of pain/discomfort.

*[Mother 25; baby 8+ weeks premature] After having a C-section, and being on a lot of medication ... I was in a wheelchair, and I had my lovely catheter bag, and everything … I managed to sit with her for five minutes, but it’s more comfortable sitting in your own room ... You’re very sore, and things.*

*[Mother 19; baby full-term] I would definitely have got less sleep if I couldn’t see her on the tablet.*

*[Father 19] And that’s not good because you need to rest, as part of the recovery process.*

Mothers who had had a baby in neonatal care previously were able to compare their experiences.

[*Mother 24; baby 15+ weeks premature] This time [with mylittleone] it felt much better, like going back up to the ward, you know like you weren’t so…I wasn’t so reluctant to leave her … So, if you had to go for painkillers or…lunch or food or anything like that it was easier to do that than you would have found it previously.*

The professionals also emphasised the importance of *mylittleone* in assisting the mothers’ recovery, and similar to the mother above, commented on the situation prior to the technology being available.

*[Professional interview 5, post-natal ward] Before [mylittleone], they would go down to the unit and they would sit there for hours and hours and they would have to really pull themselves away to come back, but now they’re coming back and having a rest in the afternoon or coming back for lunch. Before, we used to have to really chase ladies to say, you need to come back for your lunch, you know? But now, because they’ve got the [tablet], they’re quite happy to come back because their baby is almost in the room with them, really. … So, that’s helping.*

The ability of the mothers to address their own physical needs was perceived as important in aiding their early postnatal recovery, thus enabling them to better care for their babies. Use of the *mylittleone* technology was reported as being important in easing/facilitating the early process of transition to motherhood by giving the mothers time and space to take care of themselves. The ability to care of themselves whilst also monitoring their baby appeared to be important in supporting the early bonding process.

#### “Showing off” the new baby

Finally, the parents discussed the benefits that *mylittleone* afforded in allowing them to share ‘real-time’ pictures of the baby with their wider family and friends. This was important as restrictions on visiting, linked to infection control measures, meant that siblings, members of the extended family, such as grandparents, and/or friends of the family, were not permitted entry to the neonatal care unit to see the baby.

*[Mother 4; baby 5+ weeks premature] [My partner] loves [mylittleone] … just being able to see her all the time … and my mum and dad, they were up visiting yesterday and they thought it was a fantastic idea as well … because she’s in neonatal and they can’t [go in] and they can’t touch her or anything like that but it meant they didn’t have to wait to see her sort of thing.*

*[Mother 21; baby full-term] Our son was able to have a look at her on the tablet when he visited.*

*[Father 21] He was upset, because he’s only four, and he wasn’t allowed in to the neonatal unit.*

*[Mother 21] So seeing her on the tablet helped.*

Being able to introduce/show the baby off (via *mylittleone*) to their wider family and friends appeared to be important in ‘normalising’ the situation for parents. The ability to view and discuss moving images of the baby helped create a shared experience that was valued.

In sum, most parents and professionals talked about the *mylittleone* technology as a positive development in the neonatal care environment. From what was discussed, the relationship with their new baby and the parents’ emotional wellbeing appeared to have been enhanced by addressing feelings of ‘closeness’, the ability to identify and respond to the baby’s needs and by engendering a shared experience among the parents and their wider family and friends, including siblings.

### Potential disadvantages of using *mylittleone*

Importantly, while most parents believed that *mylittleone* was a positive development, for a small minority, its use had not enhanced their experience of parenting in the neonatal care environment. While they could see some benefits in its use, a few parents had decided not to use it for the duration of their hospital stay and/or would not use it again if they found themselves in a similar circumstance. The central issue was that for some, rather than providing reassurance, the ability to see the baby whenever they wanted appeared to increase anxiety levels. The themes that emerged from the parents’ and professionals’ narratives focused on: Dealing with dilemmas; Interpreting what was being seen on screen; Wondering if there was something to be concerned about; and, Parents seeing something they would rather have not.

#### Dealing with dilemmas

The following account, which is necessarily long, demonstrates some of the tensions associated with seeking to be responsive to a baby’s needs, particularly when these needs were highlighted by use of the *mylittleone* technology and might otherwise have gone unnoticed.

*[Mother 17; baby full-term] I remember at one point that I got quite upset and the reason was it was night time and I’d just been down to breastfeed him, and .......... and by the time I got back up the stairs I could see that he was crying on the screen and it really upset me … and … the staff member that was on that night, she … sort of kept putting his dummy back in and, you know, and sort of trying to shoogle the cot as it were, but you … could tell he wasn’t settling. … And it just, yeah, it really upset me because I was kind of in two minds … can I go back down again, can I not, you know, even though they’ve said that I can come and go whenever I please … would it be a case that, you know, would she not think, “Oh you’ve just been here why are you here again?”, you know, so you were in that awkward sort of stage of will I or won’t I type of thing. What I had to do was literally just put the tablet down so that I could go to sleep.*

*[Interviewer] And did you feel you could have turned the tablet off?*

*[Mother 17] Yeah but again it was almost like the sort of curiosity killed the cat, so it was like I don’t want to turn it off but I just don’t want to see it just now … And … you would almost feel like you were … like sort of not being a mother if you turned it off. You know it was almost like you don’t care enough.*

The issue of not feeling it was ‘appropriate’ to turn off the tablet device was discussed by a small number of the mothers. Concerns seemed to be linked to feeling that they were not fulfilling expectations associated with their parenting role i.e. identifying and being responsive to their baby’s needs, if they did not constantly ‘observe’ their baby.

#### Interpreting what was being seen on screen

Other concerns raised by a small number of parents, linked to their ability to make sense of what they we seeing on the tablet device.

*[Father 9; baby 6 weeks premature] It’s a double edged sword [using mylittleone], I would say.*

*[Mother 9] Yeah.*

*[Father 9] You can see what’s happening but you don’t know what’s happening. … So every time they’re doing something [to the baby], it might be routine, it either gets switched off or you see a pair of hands coming in [to the incubator/cot] with the gloves and then it gets switched off and you think, is it just something routine, is it not? And then you’d wait for maybe ten, fifteen minutes and it would come back on … and then you might see five minutes later something else happening again. As I say, you’re never quite sure* … *It wasn’t for us, no; it wasn’t for us.*

The ability of parents to interpret what they were seeing was also discussed by the professionals.

*[Professional interview 2; neoanatal unit; Participant A] I think for the anxious mums it [can] make them even more anxious*.

*[Professional interview 2; neoanatal unit; Participant B] I think a lot of the interpretation of [what they see on the tablet] and certainly where mum’s anxiety comes from, comes from mum’s experience, whether she’s a first time mum or not, whether she’s had a complicated pregnancy or not, whether there’s been pre or post anxiety or complications and things. I think that alters their interpretation of what they see on the tablet.*

Again, what was discussed demonstrates that for some, albeit a small number of parents, use of the technology was not perceived as beneficial, and in some instances, not desirable. Use of the technology appeared to give them access to information about the care of their baby that they were either not able to make sense of or they were struggling to deal with, having found themselves unexpectedly taking on the role of parent to a sick and/or premature.

#### Wondering if there is something to be concerned about

The switching off and on of the *mylittleone* camera by staff was the focus of much discussion and some debate among the parents and professionals. As noted above, when staff were undertaking a medical/nursing procedure, the policy was that the camera should be switched off. When the camera was switched off a notice appeared on the tablet device letting the parents know a procedure was underway. However, the notice was a standardised message that did not indicate what the procedure was or why it was being undertaken and there was no indication of how long the procedure would last. Procedures could vary from the changing of a nappy to resuscitation of the baby.

It was not uncommon for parents to report delays in the camera being switched back on after a procedure. For some this raised concern as they began to wonder if there was ‘something wrong’ with their baby.

*[Mother 11] One thing I didn’t like was sometimes they turned the camera off … and they would forget to turn it on again … and you had a sense of anxiousness when you couldn’t see him … I didn’t want to make a fuss and be like a neurotic mother … but I was like please can you turn it on [so I can see he’s okay].*

#### Parents seeing something they would rather have not

A final concern was that staff occasionally forgot to switch the camera off when undertaking a procedure. This meant that a small number of parents had seen procedures that had the potential to cause stress and anxiety. This issue was discussed by both the parents and professionals.

*[Mother 9; baby 6 weeks premature] Sometimes you see stuff you don’t want to see. I saw them taking blood from his heel and he was screaming the place down, he wasn’t happy.*

*[Father 9] Then he got a blockage in his oxygen tube so they started putting another tube in to suck things out.*

*[Professional interview 7; post-natal ward] I know of two instances where the mum got upset because someone had taken bloods and forgotten to turn the camera off.*

Linked to the above, there was discussion in all of the interviews with staff about what would happen in relation to the switching off of *mylittleone* in an emergency situation, where a baby required immediate attention. The scenario of an emergency clearly caused staff concern thinking about the possibility and (perceived/assumed negative) consequences of forgetting to turn off *mylittleone* as they sought to ensure the safety and well-being of the baby in their care.

In sum, while parents framed the ‘downsides’ of using *mylittleone* in different ways, there was a commonality in relation to the situations discussed. Most often the downsides related to witnessing something on screen which they otherwise would not have seen, for example, their baby crying or a medical procedure being undertaken. Importantly, some parents downplayed the significance of any negative aspects of *mylittleone* in light of their overall positive experience and other parents talked about similar events as having been more problematic for them.

Staff agreed that while for most parents, use of *mylittleone* reduced their anxiety levels, for a small number it had the opposite effect. Some had observed that it was often late at night that they would receive calls from parents about what they had seen on *mylittleone*. Similarly, they often received calls immediately the camera was switched off to undertake a procedure. The professionals felt the hyper-vigilance that could be associated with the constant ability to monitor the baby had the potential to impact negatively on the well-being of a small number of the mothers. The staff were aware that they needed to be able to identify these mothers and take appropriate action (e.g. encouraging the mothers to switch the tablet off for periods of time; asking if they wished not to use it).

The findings on the benefits and disadvantages of the *mylittleone* technology clearly demonstrate that the parents reacted differently to the same or similar events, when taking into account the health and developmental progress of their baby. Contextual factors that appears to be influential included: the health of the mother in the early post-natal period; the level of parenting experience, including experience of parenting a child in the neonatal care environment previously; and, relatedly, levels of stress and associated coping resources. For the majority, use of the technology brought with it a level of comfort and reassurance that impacted positively on their relationship with their newborn baby. However, there were two sides to the use of the new technology, for a small minority of parents *mylittleone* did not reduce and may have increased anxiety levels by providing 24/7 access to their sick and/or premature child that they did not feel they benefitted from and/or could cope with.

### Extending *mylittleone’s* reach to the home environment

At the time the interviews were conducted the *mylittleone* technology could only be used in the hospital setting. However, as it was anticipated that, in the future, video images of the baby could be transmitted to family homes when, for example, the mother had been discharged and her baby remained in hospital; parents and professionals were asked to share their views on this potential development. The two themes that emerged from the parent and professional accounts were: Going home without the baby; and, Mothers taking matters into their own hands.

#### Going home without the baby

The majority of parents felt that being able to use *mylittleone* at home would be beneficial and desirable.

*[Parent 19; baby full-term] I [am] starting to get really anxious about going home without her and thinking when I’ve been here I’ve had this camera that I’ve been able to just use all the time. Whereas at home I’m going to have absolutely nothing and I can’t imagine having like a night’s sleep. I [will] be having to phone the unit, like two or three times a night just to check on her.*

*[Mother 19] You’ve had the experience of having to go home and not being able to see her [to partner], how did you find that?*

*[Father 19] It was really tough, especially after seeing her for like the first 48 h [stayed in the hospital with mother]. It’s tough when you go home and can’t see them anymore.*

The majority of the parents had other children and they discussed the difficulty associated with being with the new baby whilst caring for older siblings. Again, the *mylittleone* technology was believed to be something that could assist.

*[Father 24; baby 15+ weeks premature] We’ve not been able to be there very much for them [older children], they have been minded by somebody else. If we were able to have mylittleone at home we would be able to work things better and they would be able to see her.*

*[Mother 24] And I wouldn’t be sat at home anxious all of the time, you could see if she was settled.*

Although parents were mostly positive about such a development, the possibility of not being able to respond to their baby’s needs from the distance of home was raised as an issue.

Staff in the post-natal ward were well-positioned to comment on the potential for *mylittleone* to be used at home as they frequently witnessed mothers being discharged before their baby.

*[Professional interview 5; post-natal ward] Ideally, the mothers would like to know that they could take it home and then see their baby from home. None of the mothers want to go home [leaving] their baby in the hospital.*

The fact that the mothers were not able to able to have regular contact via the *mylittleone* technology following their discharge was something that the professionals felt was a barrier to the continued establishment of the relationship between the parents and their baby. While parents were encouraged to visit as much as possible, the ability to connect with their baby whenever they wanted, included first thing in the morning and last thing at night, was considered to be important. The professionals also emphasised that mothers are often discharged weeks or even months in advance of their baby.

#### Mothers taking matters into their own hands

Interestingly, some of the mothers had used FaceTime® while in the post-natal care environment as a way of involving the baby’s father in seeing what they could see on the tablet device.

*[Mother 16; baby 5 weeks premature + low birth weight] [When I was in hospital] I would FaceTime® my husband [from my phone when he was at home] so he could watch her as well. So we could watch it at the same time. … Yeah, it would basically be, like, look what she’s doing now or did you see that? … And she made good improvements all the time, you know, we’d see a difference in her and she would have a bit of equipment removed and things like that and we’d just talk about her, what will happen next and how soon until she gets out and things like that.*

*[Interviewer] So it was like a wee, sort of, bonding session between the three of you?*

*[Mother 16] Yeah, [a] three way conversation, except you couldn’t talk [of course].*

Hence mothers, to an extent, had already extended the use of the *mylittleone* technology to enable their partners to ‘view’ the baby from home.

In sum, the ability to extend use of *mylittleone* to the home environment was generally viewed positively by parents and professionals. The technology appeared to have an important potential in helping parents to feel closer to their baby following the mothers’ discharge from hospital and thus to assist the ongoing transition process.

## Discussion

**Fig. 1:**
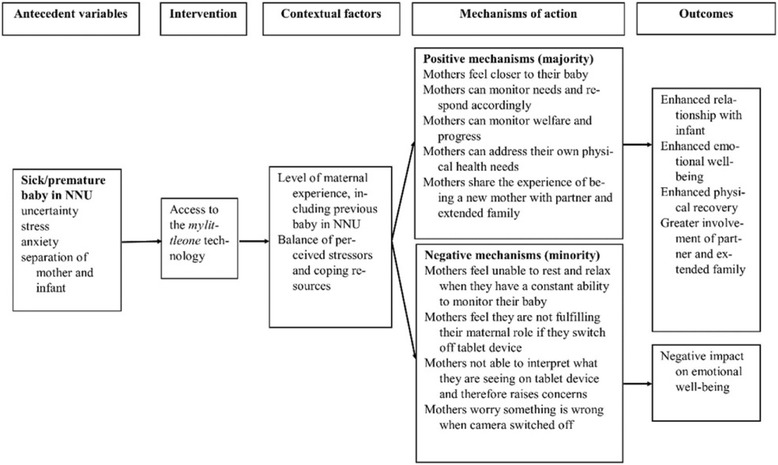
Perceived impact of *mylittleone* - empirically informed conceptual model

The current study sought to explore parent and professional views of the impact of the *mylittleone* technology, to uncover mechanisms of action, and to determine factors that appeared to influence perceptions of the technology. Based on the study results, an empirically informed conceptual model of the impact of the *mylittleone* technology was developed and is presented below (Fig. 1).

### Positive mechanisms and outcomes

As indicated, the majority of the parents and professionals who participated in the study spoke very positively about *mylittleone*, believing that it assisted the process of transition to motherhood. Perceived positive outcomes associated with its use included: an enhanced relationship with the baby; enhanced emotional wellbeing; enhanced physical recovery; and, a greater level involvement of the mother’s partner and extended family. The mechanisms of action that resulted in these perceived benefits included feelings of closeness and responsiveness engendered by the ability to see their baby on the tablet device, the constant ability to monitor the welfare and progress of their baby, the ability of the mothers to address their own physical needs, whilst monitoring their baby, and the ability to share the images of the baby, and thus the experience of being a new parent, with their partner and extended family. The perceived benefits of the *mylittleone* technology are discussed below in light of existing literature.

The developing relationship between a parent and their new-born baby is believed to be a central and important psychological process of the puerperium; however, the early establishment of the bond between the parent and infant is known to be compromised when a child is admitted to a NNU [4, 11, 30, 34]. Bonding is the term commonly used to describe the mother (and father) coming to know, love and accept her new infant and has been defined as an enduring relationship that is positive, unique to the child and occurs through the process of attachment [35, 36]. Importantly, three attributes have been identified as central to the bonding process and these are proximity, reciprocity and maternal commitment [37]. Physical proximity is required to allow a parent to bond with her baby. From what was described by the mothers and fathers in the current study, use of the *mylittleone* technology helped them to feel closer to their baby when they could not physically be with them. Reciprocity refers to the mutual/shared behaviours of the parent and infant. Again, from what was described, use of the technology allowed the mothers, in particular, to identify and respond to their infant’s needs demonstrating both reciprocity and maternal commitment. Interestingly, as noted in the Results section, engagement with the baby via the tablet device appeared to assist with milk production, encouraging the let-down reflex. While this allowed the mothers to be responsive to her babies’ needs, it is important to note that the production of breast milk is linked to levels of the hormone oxytocin and that oxytocin has also been implicated in the establishment of the maternal-infant bond [32].

As bonding and attachment have been shown to influence an infant’s emotional, cognitive and physical development, in the short and longer term, it is important that efforts are made to encourage the process [38, 39]. Findings from the current study suggest that the *mylittleone* technology may have an important contribution to make in helping to facilitate the early bonding process when periods of separation are imposed. It also appears to aid the wider transition by helping mothers be responsive to their baby’s needs. Responding to their baby’s needs is in turn likely to result in a more positive appraisal of their newly established role as mother to the infant in the NNU.

When considering maternal emotional well-being, the distress experienced by many when their child is admitted to a NNU is well-established (e.g. [8, 40]). Importantly, recent research has also demonstrated the emotional impact on fathers [12]. Parents of children who are very sick or premature often struggle with the uncertainty associated with their child’s short and longer term prognosis and the highly technical and somewhat ‘alien’ environment of the neonatal unit [41]. From what was described, the ability to see their baby was ‘stable’ when they were not with them provided the majority of parents with an important level of reassurance that, in turn, impacted positively on their emotional well-being. Use of the technology also allowed parents to prepare themselves for seeing the baby for the first time in the neonatal unit, and this was reported as helping to diminish the stress which has been shown to be associated with this event [8]. The reduction in stress and anxiety appeared to be important in enhancing the emotional well-being of the mothers and fathers and, consequently, in supporting the bonding process. The *mylittleone* technology therefore appeared to be functioning as an important coping resource, assisting the transition to parenthood.

Another perceived benefit, discussed by the majority of parents and professionals related to the scope *mylittleone* provided in assisting the mothers’ recovery. The ability that the technology provided in allowing mothers to relax and recover physically, whilst still ‘keeping in touch’ with their babies appeared to assist the bonding process. Previous research has demonstrated an association between rest and recuperate in the early post-natal period, emotional well-being, bonding and the production of breast-milk [42, 43].

Finally, parents spoke of the benefits of being able to introduce the baby (via *mylittleone*) to the wider family, including siblings and friends, who, due to issues associated with infection control, were not allowed access to the neonatal unit. This appeared to be important in normalising the situation. Previous research has shown that mothers, in particular, often feel bereft when, unlike the other mothers in the post-natal environment, they do not have a baby at their bedside to show to family members [8]. From what was discussed, the ability to see the baby may also have encouraged the number of visitors and visits. Access to existing support networks has been highlighted as important for maternal well-being in previous research and may help protect mothers who are confined to hospital from feelings of isolation [8]. In the current study, the shared experience of viewing and discussing the ‘real time’ images of the baby appeared to be important in this regard and was discussed by both the mothers and fathers.

### Negative mechanisms and outcomes

While the majority of the parents found the *mylittleone* technology to be advantageous, for a small minority of parents, the almost constant ability to see their baby that *mylittleone* provided appeared to increase rather than decrease feelings of stress and anxiety and so did not assist the process of transition to motherhood. The parents and professionals referred to what might be described as a state of hyper-vigilance i.e. a heightened level of watchfulness and feelings of protection for the baby. This form of response in parents of babies admitted to a neonatal unit has been identified in review-level evidence and generally ‘relaxes’ as the parents’ form trusting relationships with the professionals caring for their child/ren [41, 44]. However, the fact that a small number of parents decided that they did not wish to use the technology after trying it out, with others saying that they would not use the technology in the future, if they found themselves in similar circumstances, is obviously something that is important to note.

### Contextual factors influencing the maternal response

It is important to consider the factors responsible for differences in the experience of the *mylittleone* technology. When seeking to explain the wide variation in parental reactions to the same or similar events research has increasingly drawn upon models of stress and coping, with Lazarus and Folkman’s [45] theory, being one of the most commonly utilised [46]. Lazarus and Folkman [45] view stress as resulting from the transaction between an individual and the environment. The stress process is based on a number of appraisals made by the individual, in which the nature of the demands faced is compared with their perceived ability to cope. Stress is considered to be based primarily on subjective perceptions of events rather than objective circumstances. ‘Coping’ refers to an individual’s cognitive and behavioural efforts to manage the internal and external demands of the stressful situation. Coping has two major functions, that is, to reduce stressful emotions (emotion-focused coping) and to alter the demanding situation (problem-focused coping). Examples of emotion-focused coping include: distancing; escape-avoidance; exercising self-control over the expression of feelings; seeking social support and positive reappraisal. Problem-focused coping targets the cause of stress in a practical way to remove or reduce the stress. Coping is influenced not only by an individual’s appraisal of the actual demands of the situation but also by the internal and external resources s/he is able to draw upon. These resources can be categorised as material physical (e.g. maternal health in the postpartum period), psychological (e.g. beliefs, personality) and social (e.g. support from partner and family) [45].

As noted, what is described above, helps to explain why some mothers valued the use of the *mylittleone* technology and others did not. From what was described parents appraised the same or similar situations (in terms of the baby’s health and prognosis) in different ways based on the access that they had to coping resources. For example, if a mother who was particularly anxious about her baby’s health/prognosis wished to employ a coping strategy that involved ‘distancing’ herself from the situation she found herself in, the 24/7 ability that the *mylittleone* technology afforded was not perceived as beneficial. If, however, a mother who was anxious about her baby’s health/prognosis was reassured by the ability to see her baby and monitor their needs and progress, *mylittleone* functioned as a useful coping resource.

When considering the contextual factors that influenced views of the technology, maternal experience of the NNU environment previously appeared to be consistent in encouraging the mothers to compare experiences and to view *mylittleone* positively. Otherwise, views of the technology (mothers and fathers) appeared to be based on the unique balance of perceived stressors and coping resources associated with their individual circumstances.

Key, of course, is the important role that professionals have in discussing the care package offered to mothers and their partners and ensuring that it is tailored to their individual needs and preferences. Ensuring, therefore, that staff are skilled in understanding the different needs and reactions of mothers and vigilant to potentially negative outcomes is an important consideration for future implementation of this form of technology. Importantly, professionals should also consider whether difficulty in observing the baby via the technology is linked in any way to an ambivalent parenting response. In some instances, avoidance of the technology may be predictive of maladaptive coping, insecure bonding, the development of post-traumatic stress, anxiety and/or depression.

### Strengths and limitations of the study

A key strength of the current study is that it is one of few world-wide to have sought to evaluate technology to help parents feel closer to their babies when periods of separation are enforced [20]. To our knowledge, this is the first study to have undertaken an in-depth, theoretically driven, exploration of perceived outcomes. As discussed previously, a critique of the literature on the introduction of technological innovations suggests that there may be both positive and negative consequences and it is important, therefore, that clinicians and/or researchers seek to give voice to the views and experience of those for whom the technology was developed [27].

When considering methodological issues, the use of purposive sampling techniques helped to ensure heterogeneity in the parents and professionals recruited and thereby to enhance the potential transferability of the results [47]. That said, none of the parents who were recruited were from black and/or minority ethnic groups and this may limit transferability.

## Conclusion

In conclusion, we believe that this study makes a valuable contribution to the evidence base on the use of webcam technology in NNUs. To the best of our knowledge, there has, to date, been no theoretically-driven, in-depth exploration of the perceived impact of this form of technology on the parental role in the early post-natal period.

While views of the technology varied, for most (parents and professionals) it was viewed as a much needed advancement in care delivery in NNUs, functioning as an important coping resource. With a current global increase in premature births [48], webcam technology appears to offer an important solution to periods of enforced parent-infant separation in the early post-natal period.

Further work is however required, to assess cost-effectiveness. Also, if use of the technology is extended to the family home, following the mother’s discharge, and when the baby remains in hospital, it is essential that its impact in this setting is evaluated. One small scale trial undertaken, to date, found that its use in this manner was feasible and acceptable and did not reduce the number of visits made by the parents to the NNU [18].

## Abbreviations

NHS: National Health Service
NNU: Neonatal Unit
Ref: Reference
SIMD: Scottish index of multiple deprivation
UK: Uniting Kingdom
USA: United States of America
